# Supplementary material for: CRISPR/Cas9 mediated deletion of the adenosine A2A receptor enhances CAR T cell efficacy
Source: Nat Commun. 2021 May 28;12:3236. doi: 10.1038/s41467-021-23331-5 (PMC8163771; doi:10.1038/s41467-021-23331-5)
Supplement: Supplementary file 1 — Supplementary Information [file 41467_2021_23331_MOESM1_ESM.pdf]

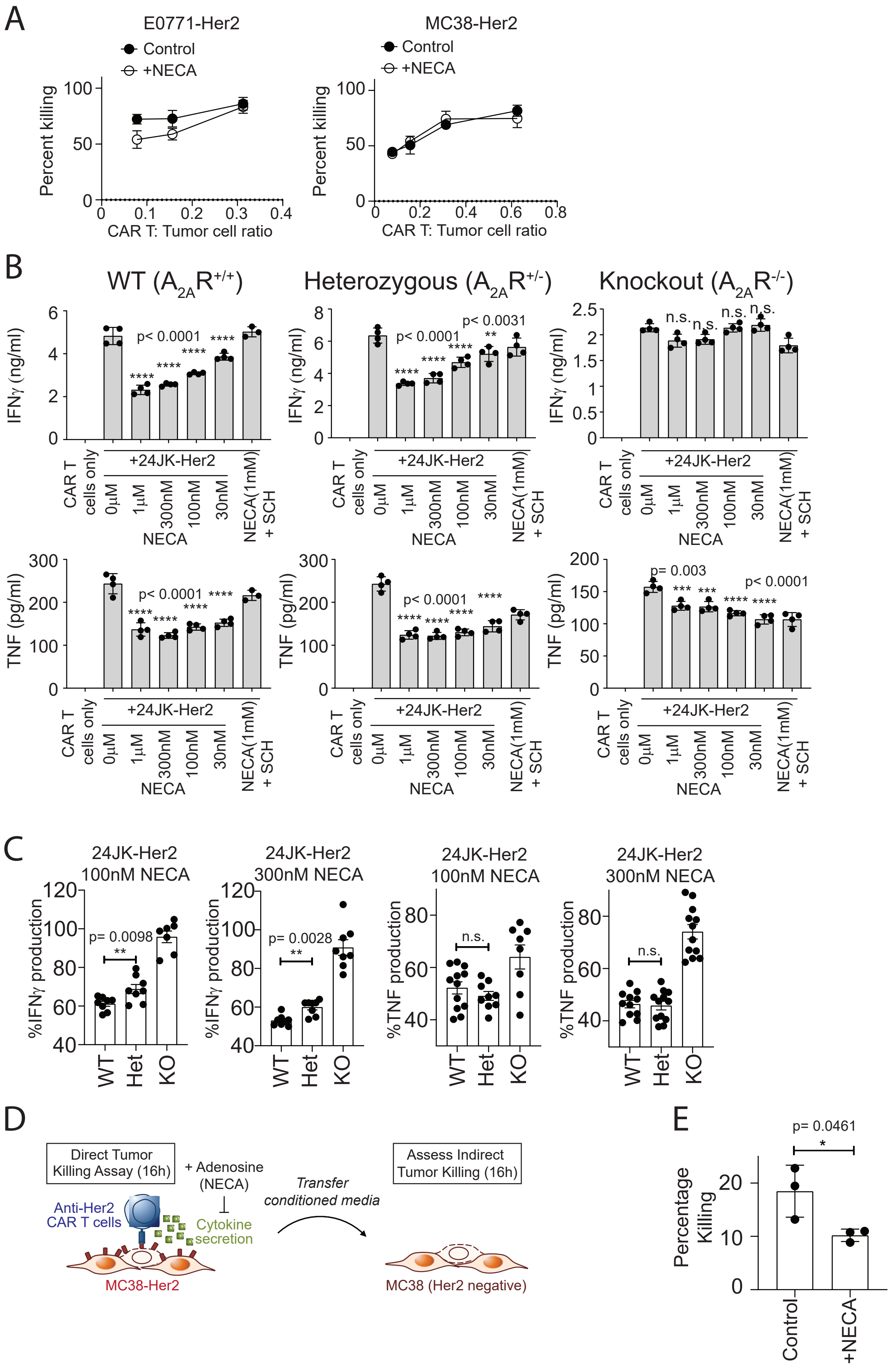

Supplementary Figure 1. $A_{2A}R$ suppresses cytokine production of anti-Her2 CAR T cells in a partially gene-dose dependent manner.

A. anti-Her2 CAR T cells were cocultured with E0771-Her2 or MC38-Her2 at the indicated CAR T: tumor cell ratios for 16 hours in the presence or absence of NECA. Data represents mean ± SD of triplicate values. **B.** 1 x 10^5 anti-Her2 CAR T cells were cocultured with 1 x 10^5 24JK-Her2 tumor cells for 16 hours in the presence or absence of SCH58261 (1 μM) or indicated concentrations of NECA. Data represents the mean ± SD of triplicate/ quadruplicate cultures (n = 4) from a representative experiment of n =2. Statistics indicate significance relative to 0 μM NECA condition. **C.** Percentage production of IFNγ and TNF relative to the control condition for each genotype. Pooled replicates from n =3 experiments is shown (IFNγ; n = 8 per group. TNF at 100nM NECA n = 12 (wild-type), 9 (heterozygous) or 8 (knockout), TNF at 300nM NECA n= 11 (wild-type) or 12 (heterozygous and knockout). B-C. **$p<0.01$, ***$p<0.001$, ****$p<0.0001$ as determined by one-way ANOVA and Tukey's post-hoc test (adjusted for multiple comparisons). Data represents the mean ± SEM. **D.** Schematic for assessment of the effect of NECA-mediated suppression of cytokine-mediated killing effect. Anti-Her2 Car T cells were cultured with MC38-Her2 at a 2:1 ratio for 8 hours in the presence or absence of NECA and supernatants then applied to MC38 cells. **E.** Percentage killing was determined by flow cytometry by quantification of the number of $7AAD^-$ live cells after 16 hours of incubation with CAR T cell supernatants. Data represented as the mean ± SD of triplicate values. *$p<0.05$ paired t test. Source data are provided as a Source Data file.

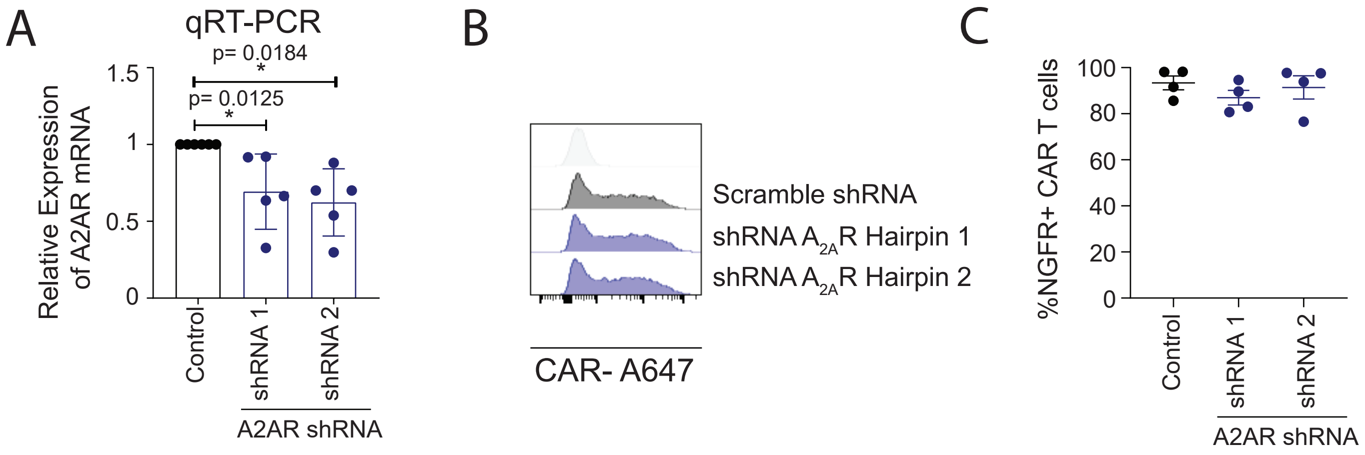

Supplementary Figure 2. Phenotype of murine A_{2A}R knockdown CAR T cells.

Anti-Her2 CAR T cells were generated with shRNAs targeting A_{2A}R or a scrambled control shRNA as per **Figure 2**. **A.** Expression of A_{2A}R mRNA as determined by qRT-PCR following isolation of $NGFR^{+}$ CAR T cells. **B.** Expression of the anti-Her2 CAR on each T cell type. **C.** Proportion of live cells expressing NGFR following MACS isolation. **A,C.** Data shown as the mean ± SEM of 4 (**C**) or 5 (**A**) experiments.*$p<0.05$ as determined by one-way ANOVA and Tukey's post-hoc test (adjusted for multiple comparisons). Source data are provided as a Source Data file.

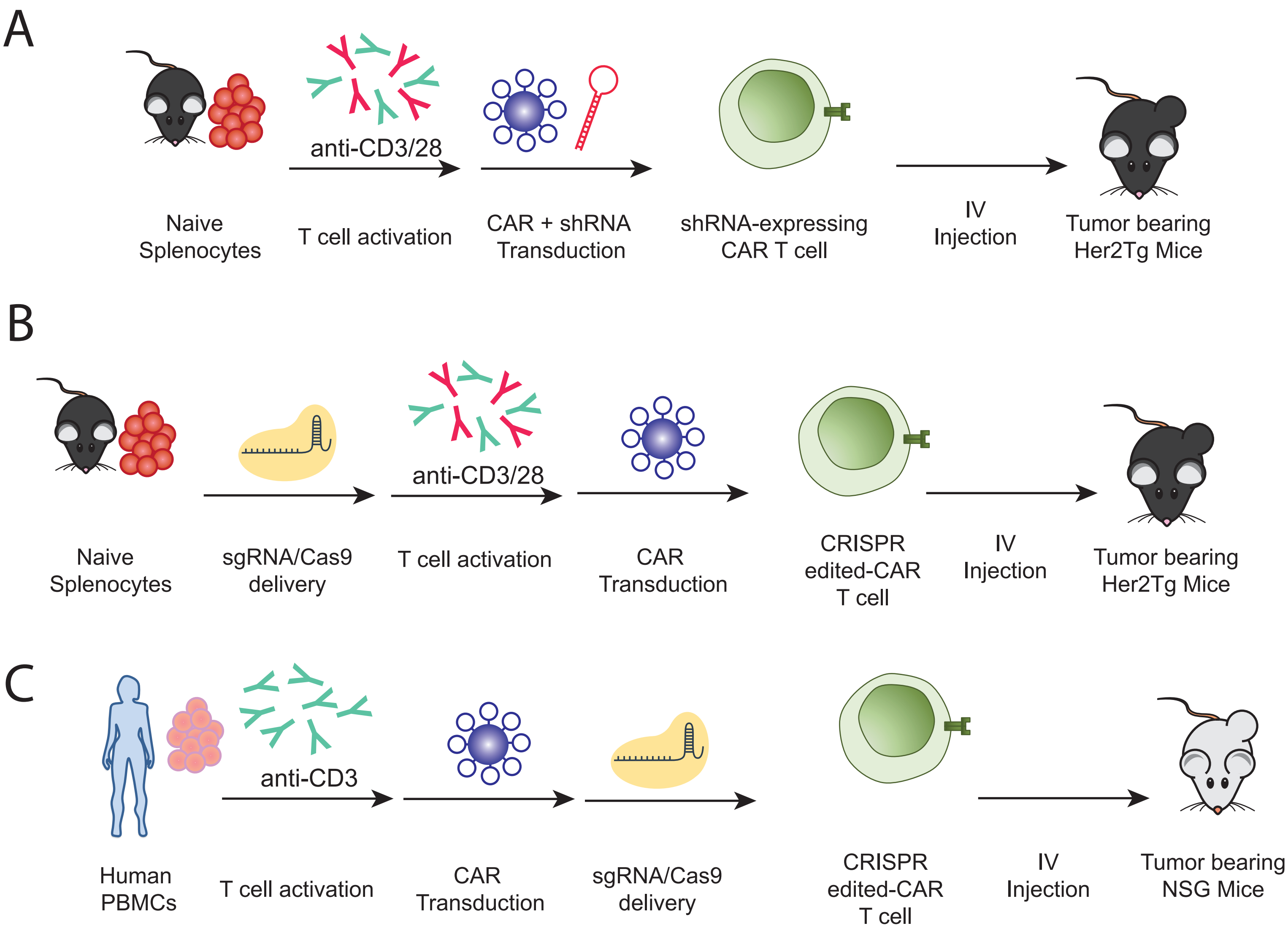

Supplementary Figure 3. Schematics for *in vivo* efficacy experiments

Experimental methodology used for assessment of *in vivo* CAR T cell function. **A.** shRNA-mediated knockdown of $A_{2A}R$ in murine anti-Her2 CAR T cells. **B.** CRISPR/Cas9 mediated targeting of the $A_{2A}R$ in murine anti-Her2 CAR T cells. **C.** CRISPR/Cas9-mediated targeting of the $A_{2A}R$ in human anti-Lewis Y CAR T cells. Source data are provided as a Source Data file.

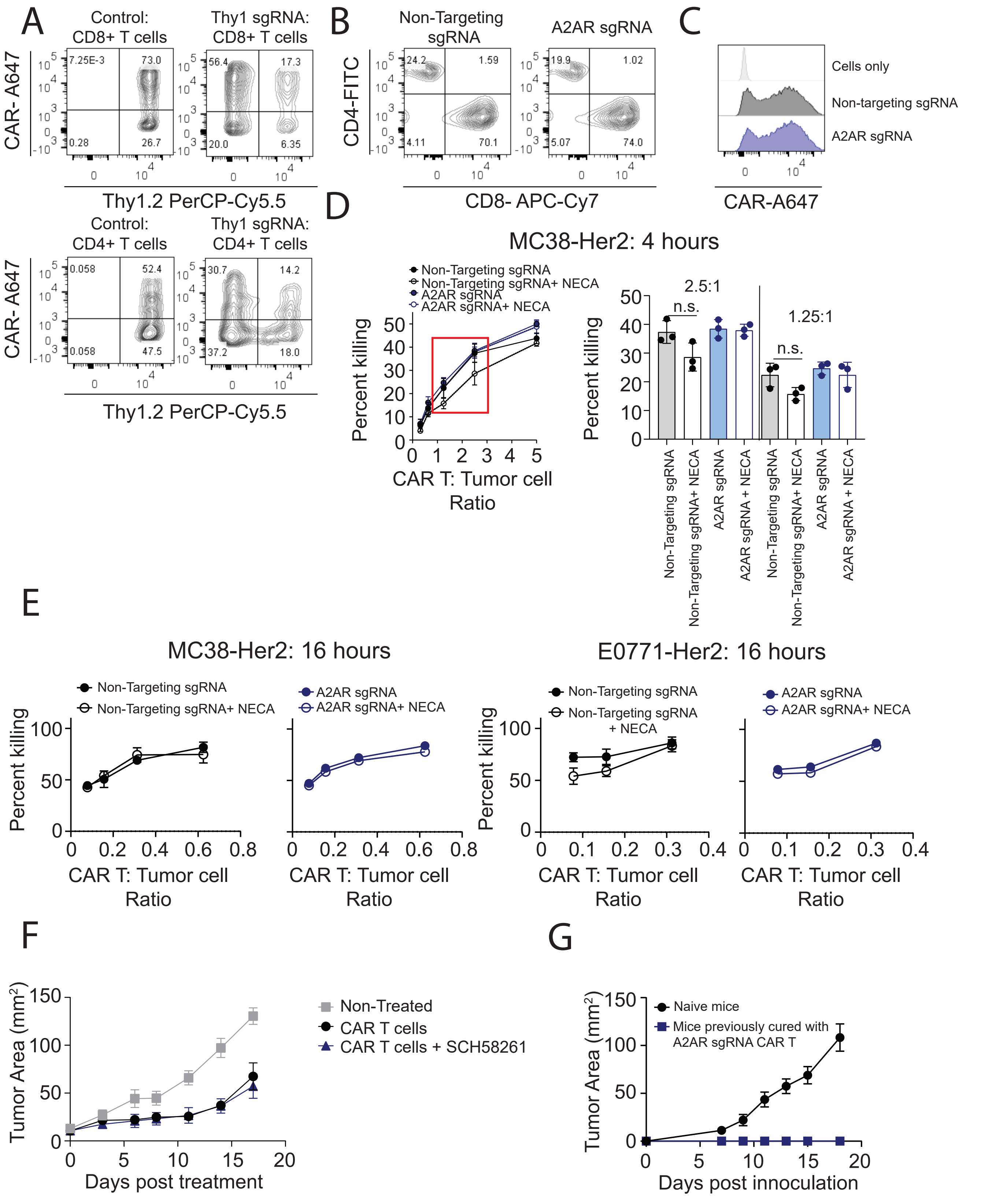

Supplementary Figure 4. Phenotype of murine CRISPR/Cas9- $A_{2A}R$ edited CAR T cells and effect of $A_{2A}R$ blockade on CAR T cell function.

A-C. Anti-Her2 CAR T cells were generated and $A_{2A}R$ or Thy1 edited using CRISPR/Cas9 as per **Figure 4**. **A**. Expression of Thy1.2 following CRISPR/Cas9 mediated editing. **B.** Expression of CD8/CD4 and **C.** the anti-Her2 CAR following CRISPR/Cas9-mediated editing of $A_{2A}R$ from a representative experiment. **D-E.** anti-Her2 CAR T cells were cocultured with MC38-Her2 or E0771-Her2 tumor cells at indicated ratios for **D.** 4 hours or **E.** 16 hours. Data represents the mean ± SD of triplicate cultures. Plots from panel **E** with control cells are the same as data presented in **Supplementary Figure 1. F.** Anti-Her2 CAR T cells were used to treat E0771-Her2 tumor bearing mice as per **Figure 3A.** Where indicated mice were dosed with 1mg/ kg SCH58261 between days 0-11 of treatment. Data is shown as the mean ± SEM of n = 5 (Non-Treated, CAR T cells) or 3 CAR T cells + SCH58261. Data from non-treated and control CAR T cell-treated mice is also presented in [1] . **G.** Mice previously bearing E0771-Her2 tumors and cured with $A_{2A}R$ edited CAR T cells were subsequently (>60 days later) injected with 2 x 10^5 E0771-Her2 cells in the fourth mammary fat pad on the opposite flank. Tumor growth was compared to naïve mice as controls. Data is presented as the mean ± SEM of 6 (naïve) or 2 (previously cured) mice per group. **D.** Statistics determined by one-way ANOVA and Tukey's post-hoc test (adjusted for multiple comparisons). Source data are provided as a Source Data file.

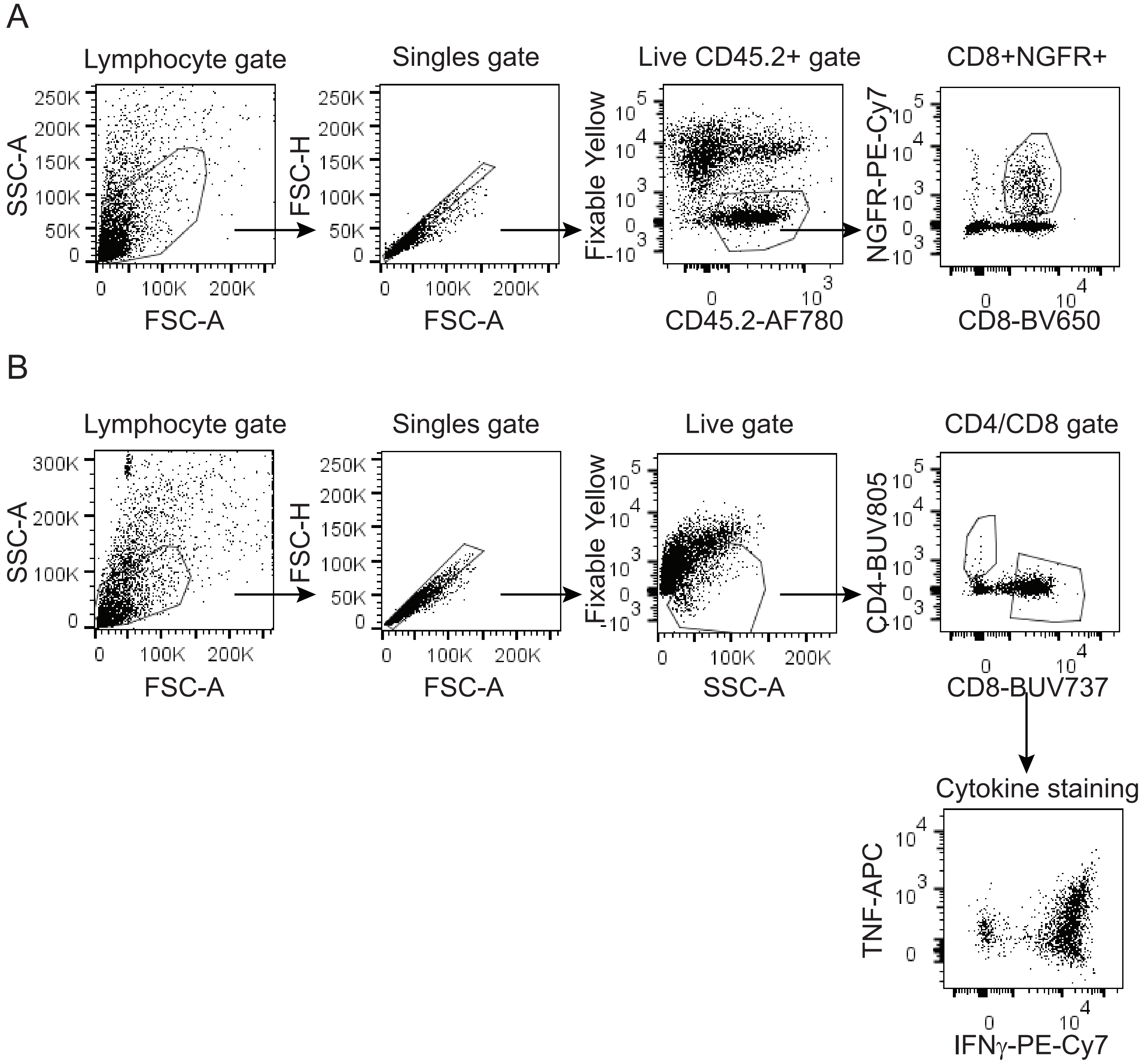

Supplementary Figure 5. Gating strategy for murine and human CAR T cells analysed ex vivo

A. Gating strategy for murine CD8^{+} CAR T cells analyzed within tumors of mice treated as per **Figure 6**. The gating strategy is morphology (lymphocytes)> single cells (excludes doublets)> CD45.2^{+}fix yellow^{-} (Viable CD45.2^{+} cells)> CD8^{+}NGFR^{+} cells **B**. Gating strategy for human CAR T cells analyzed *ex vivo* from tumors of NSG mice treated as per **Figure 8**. The indicated FACS plots are obtained from samples stimulated for 3 hours with with PMA (5 ng/ml) and ionomycin (1 µg/ml) with GolgiPlug and GolgiStop. The gating strategy is morphology (lymphocytes)> single cells (excludes doublets)> fix yellow^{-} (Viable cells)> CD8^{+} or CD4^{+} cells. A representative stain for IFNγ and TNF from the CD8^{+} gate is shown. Source data are provided as a Source Data file.

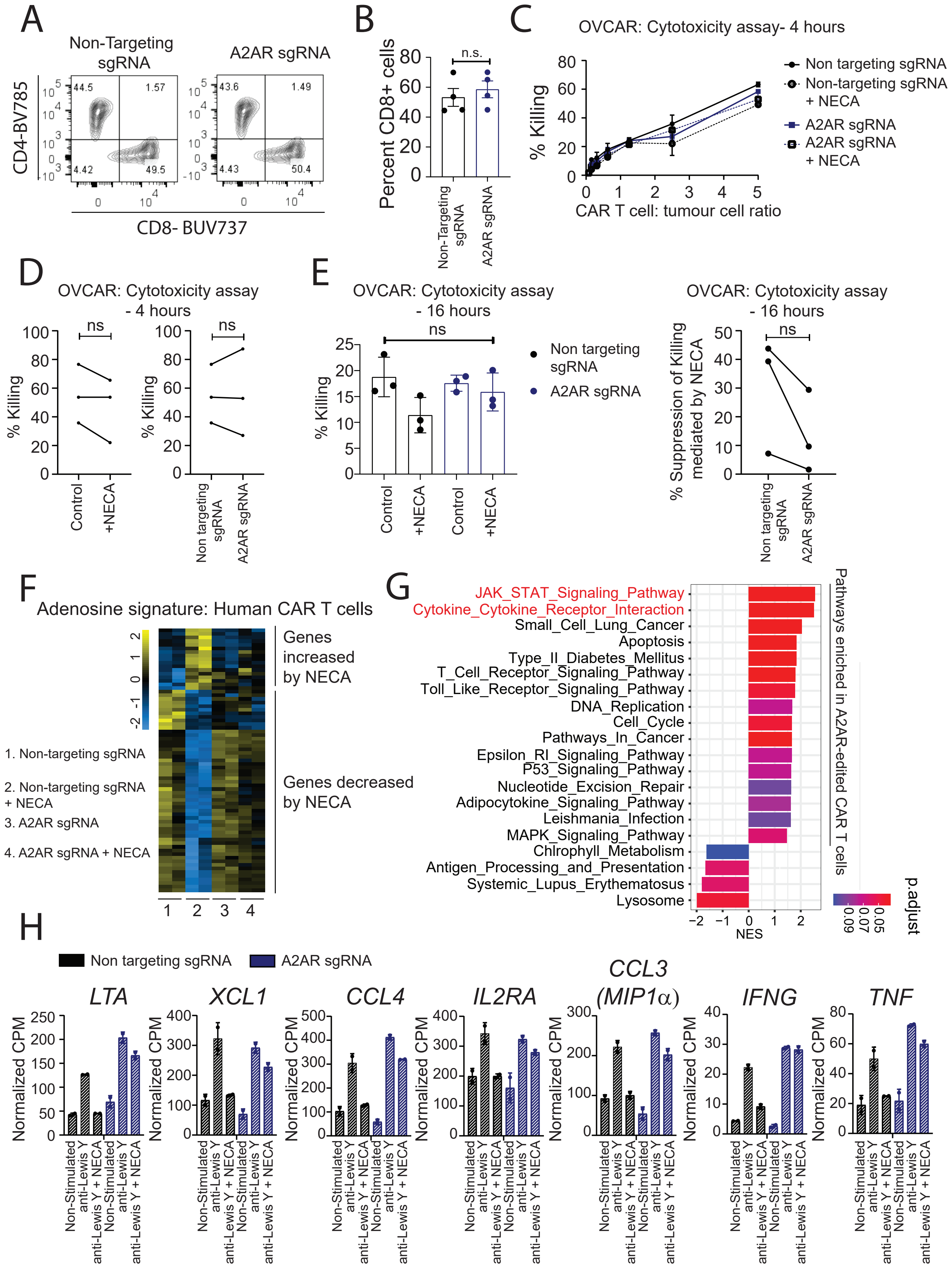

Supplementary Figure 6. Phenotype of human CRISPR/Cas9-A_{2A}R edited CAR T cells

Human anti-Lewis Y CAR T cells were generated and then treated with CRISPR/Cas9 and a sgRNA targeting either human A_{2A}R or a non-targeting control. CAR T cells were then restimulated and function determined 6-9 days post reactivation. **A-B.** Expression of CD8 and CD4 on CAR T cells. **A**. Representative donor **B.** Mean ± SEM from 4 different donors, statistics determined by paired t test.. **C-E.** CAR T cells were cocultured with 1 x 10^4 of ^{51}Cr labeled OVCAR-3 tumor cells at the indicated Effector: Target ratios for **C-D** 4 hours or **E.** 16 hours. Killing was calculated as percentage ^{51}Cr release. **C.** Data is shown as the mean ± SD of triplicate cultures **D.** Paired data from individual donors with data at a 2.5:1 Effector: Target ratio. Statistics; paired t test. **E.** Statistics; one-way ANOVA and Tukey's post-hoc test (adjusted for multiple comparisons). **F.** CAR T cells were stimulated with an anti-idiotype antibody for 8 hours in the presence or absence of NECA and RNA analyzed by 3'RNA-Seq. Heat map of expression of human adenosine signature related genes is shown. **G.** Unbiased pathway analysis for genes increased in CRISPR/Cas9 A_{2A}R-edited CAR T cells relative to control CAR T cells following activation in the presence of NECA. **H.** Normalized CPMs for cytokines/ chemokines significantly modulated by NECA identified from the KEGG pathway Cytokine: Cytokine receptor interaction. Data represented as mean ± SD of n =2 replicates. Source data are provided as a Source Data file. Statistical tests were performed with indicated R packages as outlined in Methods.

A

	Guide Binding Site	Genome Location	FWD Primer	REV Primer
On Target 1 (A2AR)	CTACTTTGTGGTGTCACTGGCGG	Chr22:24433530-24433552	CTGAAGATGGAGCTCTGCGTG	ATCATGGGCTCCTCGGTGTAC
Off Target 1 (Intergenic)	CTCTTTGTGGTGTCACTGAAGG	ChrX:36399551-36399572	GATATTCAACGTGGATGCGCCTC	CATGGAGGGCAGGATATCCAC
Off Target 2 (IFNGR1, intron 1)	CTACTTTTGGTGTCACTGAAGG	Chr6:137212145-137212166	CCCATGATTGAGCAAAGTCTCACC	CCTGGGCAACAGAGCAAGAC
Off Target 3 (OTOA, intron 12)	CTACTCTGTGGTGTCACCTGGTGG	Chr16:21711099-21711122	GCGACAGAGCAAGACTCCATTTC	CTCTCTGCAGCCCAAGATTGGA

B

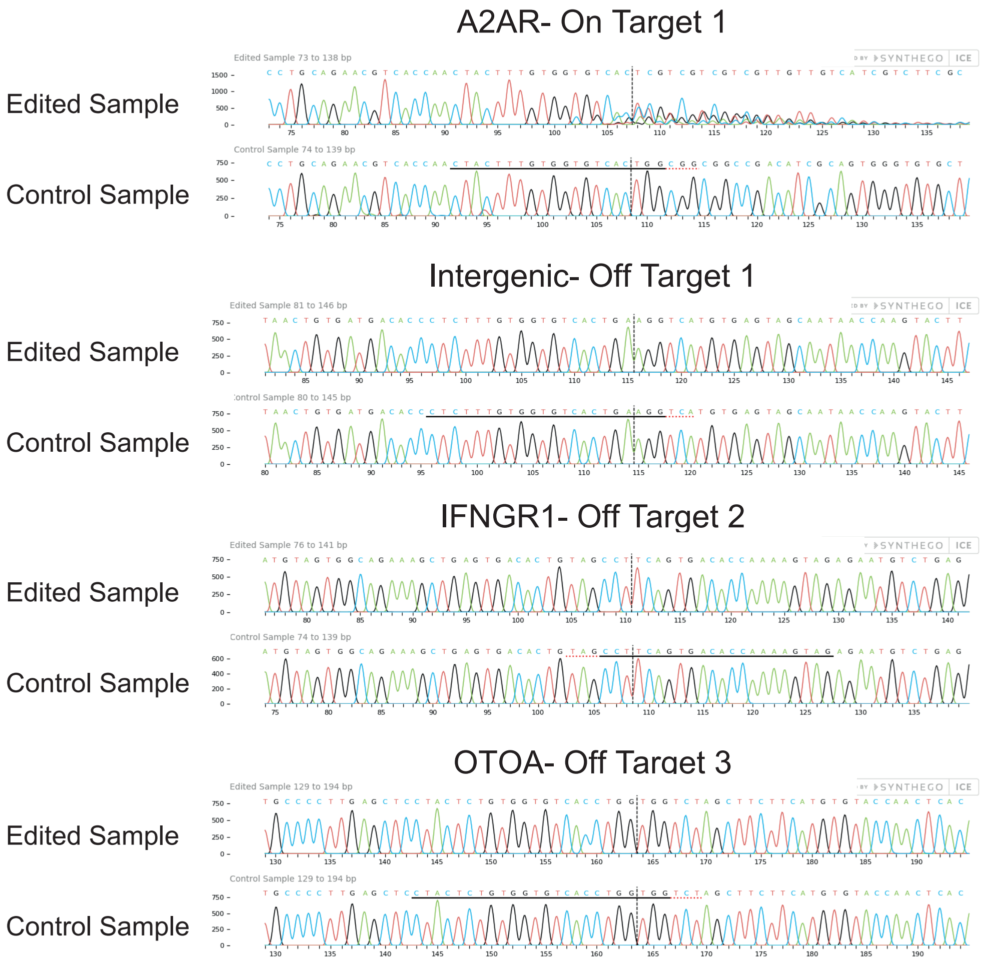

C

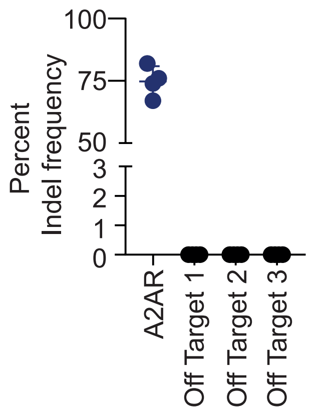

Supplementary Figure 7. Off-target editing analysis following CRISPR/Cas9 editing of human CAR T cells

Assessment of off targeted editing of the ADORA2A targeting sgRNA. **A)** COSMID analysis predicting potential off target binding sites and primers used to amplify these regions. **B)** Chromatogram summary of on target and off targeted editing in control or edited samples. Underlined regions show potential guide binding sites **C)** Summary of Indel frequencies in on target and off target binding sites as assessed by targeted Sanger sequencing and ICE analysis. Data for the $A_{2A}R$ gene replicated from **Figure 6A**. Data represented as the mean ± SEM of n = 4. Source data are provided as a Source Data file.

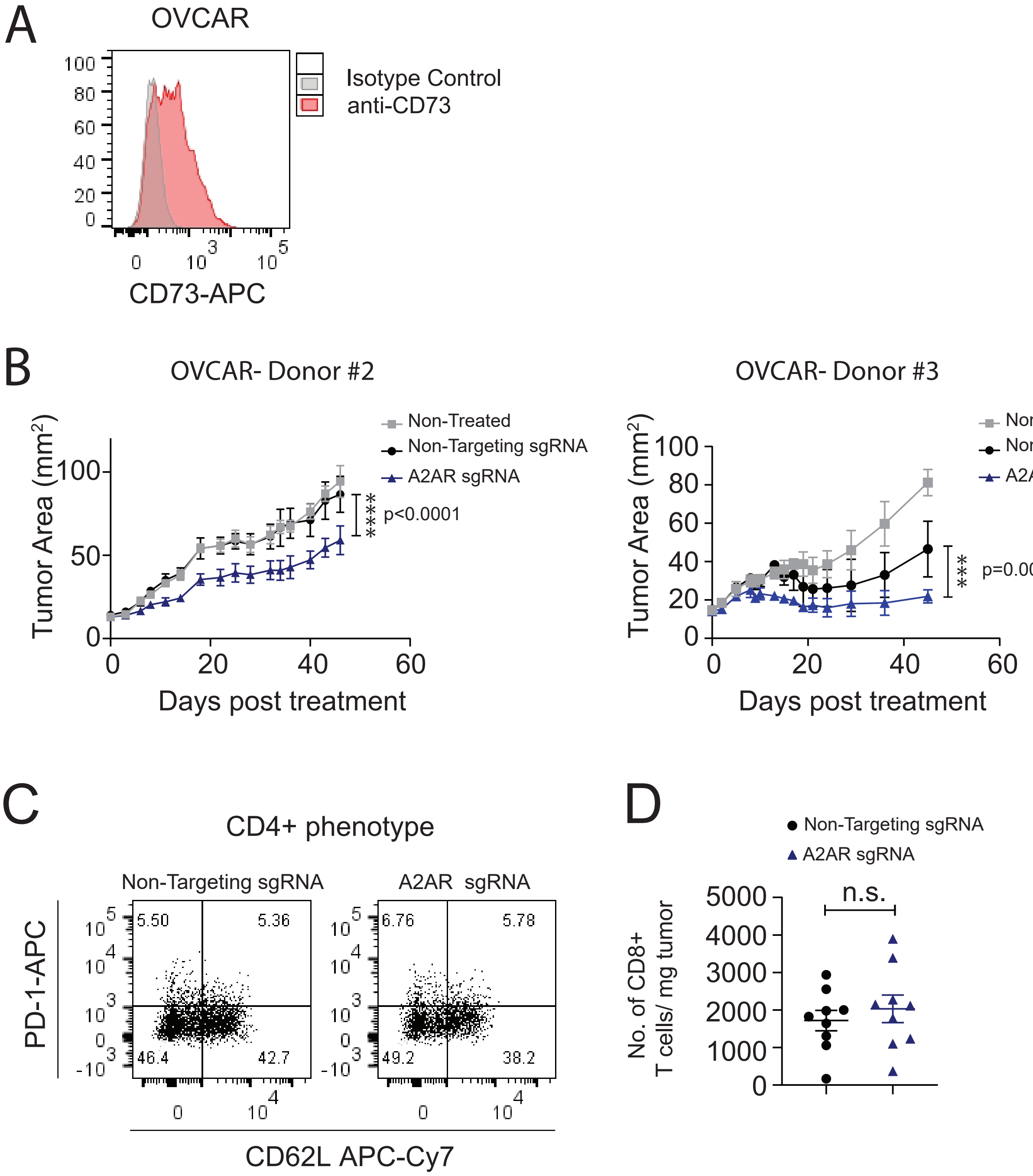

Supplementary Figure 8. Targeting $A_{2A}R$ by CRISPR/Cas9 enhances the *in vivo* efficacy of human anti-Lewis Y CAR T cells.

A. Expression of CD73 on OVCAR-3 cells. **B-D** NSG mice were injected sub-cutaneously with 5 x 10^6 OVCAR-3 tumor cells. Once tumors were established (15-20 mm^2) mice were irradiated (1 Gy) and treated with 6 x 10^6 (Donor 2) or 8 x 10^6 (Donor 3) anti-Lewis Y CAR T cells. **B.** Tumor growth for 2 individual donors n = 6 (Non-treated donor #2, mock sgRNA donor #2), 5 ($A_{2A}R$ sgRNA donor #2), 4 (non-treated donor #3) or 3 (mock sgRNA, $A_{2A}R$ sgRNA). Data shown is the mean ± SEM of 3-7 mice per group, ****p<0.0001, ***p<0.001 Two-way ANOVA. Data for non-Treated mice for Donor #2 are the same as those presented in **Figure 8A**. **C.** Mice were bled at day 8 post treatment and the phenotype of $CD4^+$ CAR T cells in terms of CD62L and PD-1 expression. Data shown is from concatenated samples (n = 7 per group). **D.** Number of $CD8^+$ CAR T cells per mg of tumor at day 14 post treatment. Data is presented as the mean ± SEM of 9 mice per group. Statistics; unpaired t test. Source data are provided as a Source Data file.

Supplementary Table 1- Antibody list

Figures	Reagent	Source	Catalog	Dilution factor
1,2,3,5	Anti-mouse CD8a, Clone 53-6.7	Biolegend	100748	1:400
1,2,3,5	Anti-mouse CD4, Clone GK1.5	BD Biosciences	612900	1:400
1,2	Anti-c-Myc Tag, clone 9B11	Cell Signalling	2233s	Neat (pellet)
1,2,3,5	Anti-mouse TNF, Clone MP6-XT22	Biolegend	506328	1:200
1,2,3,5	Anti-mouse IFN-γ, Clone XMG1.2	Biolegend	505808	1:200
1,2,3,5	Anti-mouse CD45.2, Clone 104	Biolegend	47-0454-82	1:200
1,2,3,5	Anti-mouse TCRβ, Clone H57-597	BD Biosciences	612821	1:400
1,2,3,5	Viability Fixable Yellow	Invitrogen	L34968	1:400
2,3,5	Anti-mouse TIM-3, Clone RMT3-23	Biolegend	119725	1:200
2,3,5	Anti-human NGFR, Clone ME20.4	Biolegend	345110	1:200
2,3,5	Anti-human Gzm-B Clone GB11	BD Biosciences	560213	1:100
2,3,5	Anti-human Ki-67 Clone B56	BD Biosciences	561126	1:100
2,3,5	Anti-mouse CD44, Clone IM7	BD Biosciences	565480	1:400
2,3,5	Anti-mouse PD-1, Clone 29F.1A12	Biolegend	135214	1:100
2,3,5	Anti-mouse CD62L, Clone MEL-14	BD Biosciences	564109	1:400
3,5	Anti-human/mouse IRF-4, clone 3E4	eBiosciences	11-9858-82	1:100
3,5,6,7	Flow count flourophores	Beckman Coulter	7548235F	20µL/well
5,6,7	Anti-human CD3, clone UCHT1	Biolegend	300430	1:100
5,6,7	Anti-FLAG, clone L5	Biolegend	637310	1:400
5,6,7	Anti-human CD4, clone SK3	BD biosciences	612888	1:400
5,6,7	Anti-human CD8, clone SK1	BD biosciences	612754	1:400
5,6,7	Anti-human CD69, clone FN50	Biolegend	310926	1:100
5,6,7	Anti-human TIM-3, clone F38-2E2	Biolegend	345008	1:100
5,6,7	Anti-human IFNγ, clone 45.B3	BD Biosciences	557844	1:100
5,6,7	Anti-human TNF	BD Biosciences	551384	1:100
5,6,7	Anti-human CD4, clone OKT4	Biolegend	317442	1:400
5,6,7	Anti-human CD62L, clone DREG-56	Biolegend	304814	1:100
5,6,7	Anti-human PD-1, clone EH12.2H7	Biolegend	329910	1:100
5,6,7	Anti-human CD27, clone O323	Biolegend	302834	1:100
5,6,7	Anti-human CD45RA, clone HI100	Biolegend	304130	1:100
5,6,7	Anti-human CD45RO, clone UCHL1	Biolegend	304212	1:100
Supp	Anti-human CD73, clone AD2	BD Biosciences	580847	1:100

References

1. Giuffrida, L. et al. IL-15 Preconditioning Augments CAR T Cell Responses to Checkpoint Blockade for Improved Treatment of Solid Tumors. Mol Ther 28, 2379-2393 (2020).
