# Supplementary material for: CRISPR/Cas9 mediated deletion of the adenosine A2A receptor enhances CAR T cell efficacy
Source: Nat Commun. 2021 May 28;12:3236. doi: 10.1038/s41467-021-23331-5 (PMC8163771; doi:10.1038/s41467-021-23331-5)
Supplement: Supplementary file 3 — Descriptions of Additional Supplementary Files [file 41467_2021_23331_MOESM3_ESM.pdf]

Descriptions of Additional Supplementary Files

Supplementary data 1
Description: Adenosine signature in murine CAR T cells.

Supplementary data 2
Description: Adenosine signature in human CAR T cells.
