# Supplementary material for: CRISPR/Cas9 mediated deletion of the adenosine A2A receptor enhances CAR T cell efficacy
Source: Nat Commun. 2021 May 28;12:3236. doi: 10.1038/s41467-021-23331-5 (PMC8163771; doi:10.1038/s41467-021-23331-5)
Supplement: Supplementary file 6 — Reporting Summary [file 41467_2021_23331_MOESM6_ESM.pdf]

nature research

Corresponding author(s): Paul A. Beavis, Phillip K Darcy, Imran G. House

Last updated by author(s): 2021-30-03

Reporting Summary

Nature Research wishes to improve the reproducibility of the work that we publish. This form provides structure for consistency and transparency in reporting. For further information on Nature Research policies, see our Editorial Policies and the Editorial Policy Checklist.

Statistics

For all statistical analyses, confirm that the following items are present in the figure legend, table legend, main text, or Methods section.

n/a	Confirmed	
☐	☒	The exact sample size (*n*) for each experimental group/condition, given as a discrete number and unit of measurement
☐	☒	A statement on whether measurements were taken from distinct samples or whether the same sample was measured repeatedly
☐	☒	The statistical test(s) used AND whether they are one- or two-sided<br>*Only common tests should be described solely by name; describe more complex techniques in the Methods section.*
☐	☒	A description of all covariates tested
☐	☒	A description of any assumptions or corrections, such as tests of normality and adjustment for multiple comparisons
☐	☒	A full description of the statistical parameters including central tendency (e.g. means) or other basic estimates (e.g. regression coefficient) AND variation (e.g. standard deviation) or associated estimates of uncertainty (e.g. confidence intervals)
☐	☒	For null hypothesis testing, the test statistic (e.g. *F*, *t*, *r*) with confidence intervals, effect sizes, degrees of freedom and *P* value noted<br>*Give P values as exact values whenever suitable.*
☒	☐	For Bayesian analysis, information on the choice of priors and Markov chain Monte Carlo settings
☒	☐	For hierarchical and complex designs, identification of the appropriate level for tests and full reporting of outcomes
☒	☐	Estimates of effect sizes (e.g. Cohen's *d*, Pearson's *r*), indicating how they were calculated

Our web collection on statistics for biologists contains articles on many of the points above.

Software and code

Policy information about availability of computer code

Data collection	Flow Cytometry: BD FacsDiva version 8 software (FlowJo LLC), cAMP assays: Wallac Envision Manager<br>Microscopy: Olympus VS120 microscope (H &E).
Data analysis	Flow Cytometry: FlowJo (Version 10.5.0), FCAP array (version 3), Statistical analysis (Graphpad Prism (Version 8.0.2), Nanostring (nsolver 4.0), Gene Set enrichment analysis: Analysis of KEGG pathways: Bioconductor R package 'fgsea' Fgsea v3.12. RNA-seQC- VERSION: v1.1.8.1. INDEL analysis: ICE (https://ice.synthego.com; version 1). COSMID tool (https://crispr.bme.gatech.edu/; Version 1). Wallac Envision Manager v1.12 software.

For manuscripts utilizing custom algorithms or software that are central to the research but not yet described in published literature, software must be made available to editors and reviewers. We strongly encourage code deposition in a community repository (e.g. GitHub). See the Nature Research guidelines for submitting code & software for further information.

Data

Policy information about availability of data

All manuscripts must include a data availability statement. This statement should provide the following information, where applicable:

- Accession codes, unique identifiers, or web links for publicly available datasets
- A list of figures that have associated raw data
- A description of any restrictions on data availability

The RNA-sequencing data that support the findings of this study have been deposited in GEO NCBI under the accession code GSE156192 that contains the sub-series GSE156189, GSE156190, GSE156191 and GSE166807. The remaining data is available within the article, supplementary information and tables or available upon request from the authors. KEGG datasets utilized can be accessed via https://www.gsea-msigdb.org/gsea/msigdb/index.jsp.

Field-specific reporting

Please select the one below that is the best fit for your research. If you are not sure, read the appropriate sections before making your selection.

- [x] Life sciences
- [ ] Behavioural & social sciences
- [ ] Ecological, evolutionary & environmental sciences

For a reference copy of the document with all sections, see nature.com/documents/nr-reporting-summary-flat.pdf

Life sciences study design

All studies must disclose on these points even when the disclosure is negative.

Sample size	Experiments were designed with sufficient power to achieve statistical significance based upon an effect size of 30%. All therapeutic experiments were performed with a minimum of n = 5 per group and replicated for at least 2 individual, independent experiments
Data exclusions	No data were excluded from the manuscript
Replication	All attempts at replication were successful. Refer to figure legends for more information
Randomization	Mice were randomized prior to treatment according tumor size to ensure all groups had equivalent tumor burden prior to the onset of therapy. All groups were age and sex matched. For in vitro studies randomisation was not required since a homogenous pool of tumour cells and/or T cells were used with the indicated conditions.
Blinding	The investigators performed, acquired and analyzed the experiments and as such were not blinded. The same investigators were involved in the randomisation, treatment (including daily treatment relevant to each treatment group) and analysis of experiments and so blinding was not possible.

Reporting for specific materials, systems and methods

We require information from authors about some types of materials, experimental systems and methods used in many studies. Here, indicate whether each material, system or method listed is relevant to your study. If you are not sure if a list item applies to your research, read the appropriate section before selecting a response.

Materials & experimental systems

n/a	Involved in the study	
[ ]	[x]	Antibodies
[ ]	[x]	Eukaryotic cell lines
[x]	[ ]	Palaeontology and archaeology
[ ]	[x]	Animals and other organisms
[x]	[ ]	Human research participants
[x]	[ ]	Clinical data
[x]	[ ]	Dual use research of concern

Methods

n/a	Involved in the study	
[x]	[ ]	ChIP-seq
[ ]	[x]	Flow cytometry
[x]	[ ]	MRI-based neuroimaging

Antibodies

Antibodies used

All antibodies used in this study were obtained from commercial suppliers (BD Pharmingen, Cell Signalling, eBIoscience, Invitrogen, Thermo Scientific, or Biolegend). A list of relevant information on the antibodies (Supplier, Catalog number, clone, dilution, lot number, technical data or QC sheet where available is provided in a Supplementary Table.
Anti-mouse CD8a, Clone 53-6.7 Biolegend 100748 1:400
Anti-mouse CD4, Clone GK1.5 BD Biosciences 612900 1:400
Anti-c-Myc Tag, clone 9B11 Cell Signalling 2233s Neat (pellet)
Anti-mouse TNF, Clone MP6-XT22 Biolegend 506328 1:200
Anti-mouse IFN-γ, Clone XMG1.2 Biolegend 505808 1:200
Anti-mouse CD45.2, Clone 104 Biolegend 47-0454-82 1:200
Anti-mouse TCRβ, Clone H57-597 BD Biosciences 612821 1:400
Viability Fixable Yellow Invitrogen L34968 1:400
Anti-mouse TIM-3, Clone RMT3-23 Biolegend 119725 1:200
Anti-human NGFR, Clone ME20.4 Biolegend 345110 1:200
Anti-human Gzm-B Clone GB11 BD Biosciences 560213 1:100
Anti-human Ki-67 Clone B56 BD Biosciences 561126 1:100
Anti-mouse CD44, Clone IM7 BD Biosciences 565480 1:400
Anti-mouse PD-1, Clone 29F.1A12 Biolegend 135214 1:100
Anti-mouse CD62L, Clone MEL-14 BD Biosciences 564109 1:400
Anti-human/mouse IRF-4, clone 3E4 eBiosciences 11-9858-82 1:100

Flow couunt flourophores Beckman Coulter 7548235F 20µL/well
Anti-human CD3, clone UCHT1 Biolegend 300430 1:100
Anti-FLAG, clone L5 Biolegend 637310 1:400
Anti-human CD4, clone SK3 BD biosciences 612888 1:400
Anti-human CD8, clone SK1 BD biosciences 612754 1:400
Anti-human CD69, clone FN50 Biolegend 310926 1:100
Anti-human TIM-3, clone F38-2E2 Biolegend 345008 1:100
Anti-human IFNγ, clone 45.B3 BD Biosciences 557844 1:100
Anti-human TNF BD Biosciences 551384 1:100
Anti-human CD4, clone OKT4 Biolegend 317442 1:400
Anti-human CD62L, clone DREG-56 Biolegend 304814 1:100
Anti-human PD-1, clone EH12.2H7 Biolegend 329910 1:100
Anti-human CD27, clone O323 Biolegend 302834 1:100
Anti-human CD45RA, clone HI100 Biolegend 304130 1:100
Anti-human CD45RO, clone UCHL1 Biolegend 304212 1:100
Anti-human CD73, clone AD2 BD Biosciences 580847 1:100

Validation

Anti-mouse CD8a, Clone 53-6.7 "FC - Quality tested, Each lot of this antibody is quality control tested by immunofluorescent staining withflow cytometric analysis. For immunofluorescent staining using the µg size, thesuggested use of this reagent is ≤0.5 µg per million cells in 100 µl volume. Forimmunofluorescent staining using µl sizes, the suggested use of this reagent is 5 µlper million cells in 100 µl staining volume or 5 µl per 100 µl of whole blood. It isrecommended that the reagent be titrated for optimal performance for eachapplication."

Anti-mouse CD4, Clone GK1.5 Two color flow cytometric analysis of CD4 expression on mouse splenocytes. Mouse splenic leucocytes were preincubated with Purified Rat Anti-Mouse CD16/32 antibody (Mouse Fc Block™)(Cat. No. 553141/553142). The cells were then stained with APC Hamster Anti-Mouse CD3e antibody (Cat. No. 553066/561826) and either BD Horizon™ BUV805 Rat IgG2b, κ Isotype Control (Cat. No. 612762; Left Plot) or BD Horizon™ BUV805 Rat Anti-Mouse CD4 antibody (Cat. No. 612900; Right Plot) at 0.25 µg/test. The two-color fluorescence contour plot showing the correlated expression of CD4 (or Ig Isotype control staining) versus CD3e were derived from gated events with the forward and side light-scatter characteristic of viable splenic leucocytes. Flow cytometry and data analysis were performed using a BD LSRFortessa™ X-20 Cell Analyzer System and FlowJo™ software.

Anti-c-Myc Tag, clone 9B11 Flow cytometric analysis of COS cells, untransfected (blue) or transfected with Myc-tagged Akt (green), using Myc-Tag (9B11) Mouse mAb (Alexa Fluor® 647 Conjugate).

Anti-mouse TNF, Clone MP6-XT22 "ICFC - Quality tested, Each lot of this antibody is quality control tested by intracellular immunofluorescentstaining with flow cytometric analysis. For flow cytometric staining using the µg size,the suggested use of this reagent is ≤0.25 µg per million cells in 100 µl volume. For flow cytometric staining using the µl size, the suggested use of this reagent is 5 µl per million cells in 100 µl staining volume or 5 µl per 100 µl of whole blood. It isrecommended that the reagent be titrated for optimal performance for eachapplication."

Anti-mouse IFN-γ, Clone XMG1.2, PE "ICFC - Quality tested, Each lot of this antibody is quality control tested by intracellular immunofluorescentstaining with flow cytometric analysis. For flow cytometric staining, the suggesteduse of this reagent is ≤0.25 µg per million cells in 100 µl volume. It is recommendedthat the reagent be titrated for optimal performance for each application."
Anti-mouse CD45.2, Clone 104 Applications Reported: This 104 antibody has been reported for use in flow cytometric analysis. Applications Tested: This 104 antibody has been tested by flow cytometric analysis of mouse splenocytes. This can be used at less than or equal to 1 µg per test. A test is defined as the amount (µg) of antibody that will stain a cell sample in a final volume of 100 µL. Cell number should be determined empirically but can range from 10^5 to 10^8 cells/test. It is recommended that the antibody be carefully titrated for optimal performance in the assay of interest.

Anti-mouse TCRβ, Clone H57-597 Two-color flow cytometric analysis of TCRβ expression on mouse splenocytes. Mouse splenic leucocytes were preincubated with Purified Rat Anti-Mouse CD16/CD32 antibody (Mouse BD Fc Block™) (Cat. No. 553141/553142). The cells were then stained with PE Rat Anti-Mouse CD4 (Cat. No. 553048/553049/561837) and PE Rat Anti-Mouse CD8a (Cat. No. 553033/553032/561095) antibodies and either BD Horizon™ BUV737 Hamster IgG2, λ1 Isotype Control (Cat. No. 612822; Left Plot) or BD Horizon BUV737 Hamster Anti-Mouse TCR β Chain antibody (Cat. No. 612821; Right Plot) at 0.5 µg/test. BD Via-Probe™ Cell Viability 7-AAD Solution (Cat. No. 555815/555816) was added to cells right before analysis. The two-color flow cytometric dot plot showing the correlated expression of TCRβ (or Ig Isotype control staining) versus CD4 and CD8a was derived from gated events with the forward and side light-scatter characteristics of viable (7-AAD-negative) splenic leucocytes. Flow cytometry and data analysis were performed using a BD LSRFortessa™ Cell Analyzer System and FlowJo™ software.

Anti-mouse TIM-3, Clone RMT3-23, BV785 "FC- Quality tested, Each lot of this antibody is quality control tested by immunofluorescent staining withflow cytometric analysis. For flow cytometric staining, the suggested use of this reagent is ≤ 0.25 µg per million cells in 100 µl volume. It is recommended that thereagent be titrated for optimal performance for each application."
Anti-human NGFR, Clone ME20.4, PEcy7 "FC- Quality tested, e Each lot of this antibody is quality control tested by immunofluorescent staining withflow cytometric analysis. For flow cytometric staining, the suggested use of this reagent is 5 µl per million cells in 100 µl staining volume or 5 µl per 100 µl of wholeblood."

Anti-human Gzm-B Clone GB11 AF700 Expression of granzyme B by peripheral blood CD8+ lymphocytes. Whole human blood was lysed with BD Pharm Lyse™ Lysing Buffer (Cat No. 555899) prior to staining with GB11. Whole lysed human blood was subsequently fixed, permeabilized and stained with mouse anti-human granzyme B antibody (Alexa Fluor® 700 GB11, Cat. No. 560213), gated on a positive CD8+ lymphocytes population. To demonstrate the specificity of this staining, the binding of Alexa Fluor® 700 GB11 was

blocked by preincubation of the fixed/permeabilized cells with excess of an unlabelled GB11 antibody (10 μg, data not shown) prior to staining.

Anti-human Ki-67 Clone B56 AF647 Flow cytometric analysis of Ki-67 expression by proliferating Jurkat and noncycling human peripheral blood mononuclear cells (PBMC). Jurkat and PBMC were fixed and permeabilized with 70% ice cold ethanol, washed, and stained with Alexa Fluor® 647 Mouse Anti-Ki-67 antibody (Cat. No. 561126) according to the BD Biosciences support protocol, Flow Cytometry Staining Protocol for Detection of Ki-67. The cells were then RNase A (Sigma Cat. No. R-5500) treated and counterstained with Propidium Iodide Staining Solution (Cat. No. 556463) to stain DNA . Flow cytometry was performed using a BD LSR™ II flow cytometry system.

Anti-mouse CD44, Clone IM7, APCR700 Flow cytometric analysis of CD44 expression on mouse bone-marrow cells. Mouse bone-marrow cells were preincubated with Purified Rat Anti-Mouse CD16/CD32 antibody (Mouse BD Fc Block™) (Cat. No. 553141/553142). The cells were then stained with either BD Horizon™ APC-R700 Rat IgG2b, κ Isotype Control (Cat. No. 564984; dashed line histogram) or BD Horizon APC-R700 Rat Anti-Mouse CD44 antibody (Cat. No. 565480; solid line histogram). Flow cytometric analysis was performed using a BD LSRFortessa™ Cell Analyzer System.

Anti-mouse PD-1, Clone 29F.1A12, FITC "FC - Quality tested, Each lot of this antibody is quality control tested by immunofluorescent staining withflow cytometric analysis. For flow cytometric staining, the suggested use of thisreagent is ≤1.0 μg per million cells in 100 μl volume. It is recommended that thereagent be titrated for optimal performance for each application."

Anti-mouse CD62L, Clone MEL-14, BV786 "FC- Routinely tested. For optimal and reproducible results, BD Horizon Brilliant Stain Buffer should be used anytime two or more BD Horizon Brilliant dyes are used in thesame experiment. Fluorescent dye interactions may cause staining artifacts which may affect data interpretation. The BD Horizon Brilliant Stain Bufferwas designed to minimize these interactions. More information can be found in the Technical Data Sheet of the BD Horizon Brilliant Stain Buffer (Cat.No. 563794/566349) or the BD Horizon Brilliant Stain Buffer Plus (Cat. No. 566385)."

Anti-human/mouse IRF-4, clone 3E4, FITC "Applications Reported: This 3E4 antibody has been reported for use in intracellular staining followed by flow cytometric analysis. Applications Tested: This 3E4 antibody has been tested by intracellular staining and flow cytometric analysis of stimulated normal human peripheral blood cells using the Foxp3/Transcription Factor Staining Buffer Set (cat 00-5523) and protocol. Please see Best Protocols Section (Staining intracellular Antigens for Flow Cytometry) for staining protocol (refer to Protocol B: One-step protocol for intracellular (nuclear) proteins). This can be used at less than or equal to 0.125 μg per test. A test is defined as the amount (μg) of antibody that will stain a cell sample in a final volume of 100 μL. Cell number should be determined empirically but can range from 10^5 to 10^8 cells/test. It is recommended that the antibody be carefully titrated for optimal performance in the assay ofinterest."

Anti-human CD3, clone UCHT1, Percpcy5.5 "FC - Quality tested, Each lot of this antibody is quality control tested by immunofluorescent staining withflow cytometric analysis. For flow cytometric staining, the suggested use of this reagent is 5 μl per million cells in 100 μl staining volume or 5 μl per 100 μl of wholeblood."

Anti-FLAG, clone L5, PE "FC, ICFC - Quality tested, Each lot of this antibody is quality control tested by immunofluorescent staining withflow cytometric analysis. For flow cytometric staining, the suggested use of thisr eagent is ≤0.125 μg per million cells in 100 μl volume. It is recommended that the reagent be titrated for optimal performance for each application."

Anti-human CD4, clone SK3, BUV805 Flow cytometric analysis of CD4 expression on human peripheral blood lymphocytes. Human whole blood was stained with either BD Horizon™ BUV805 Mouse IgG1, κ Isotype Control (Cat. No. 612897; dashed line histogram) or BD Horizon BUV805 Mouse Anti-Human CD4 antibody (Cat. No. 612887/612888; solid line histogram). The erythrocytes were lysed with BD FACS™ Lysing Solution (Cat. No. 349202). Flow cytometry and data analysis were performed using a BD LSRFortessa™ X-20 Cell Analyzer System and FlowJo™ software.

Anti-human CD8, clone SK1, BUV737 Flow cytometric analysis of CD8 expression on human peripheral blood lymphocytes. Human whole blood was stained with either BD Horizon™ BUV737 Mouse IgG1, κ Isotype Control (Cat. No. 612758; dashed line histogram) or BD Horizon BUV737 Mouse Anti-Human CD8 antibody (Cat. No. 612754/612755; solid line histogram). The erythrocytes were lysed with BD FACS™ Lysing Solution (Cat. No. 349202). Flow cytometry and data analysis were performed using a BD LSRFortessa™ X-20 Cell Analyzer System and FlowJo™ software.

Anti-human CD69, clone FN50, Percpcy5.5 "FC - Quality tested, Each lot of this antibody is quality control tested by immunofluorescent staining withflow cytometric analysis. For flow cytometric staining, the suggested use of this reagent is 5 μl per million cells in 100 μl staining volume or 5 μl per 100 μl of whole blood."

Anti-human TIM-3, clone F38-2E2, BV421 "FC - Quality tested, Each lot of this antibody is quality control tested by immunofluorescent staining withflow cytometric analysis. For flow cytometric staining, the suggested use of this reagent is 5 μl per million cells in 100 μl staining volume or 5 μl per 100 μl of whole blood."

Mouse Anti-human IFNγ, clone 45.B3, PEcy7 Expression of IFN-γ by stimulated human peripheral blood lymphocytes. Human PBMC were stimulated for 6 hours with PMA (50 ng/ml final concentration; Sigma) and calcium ionophore A23187 (500 ng/ml final concentration; Sigma) in the presence of GolgiStop™ (2 μM final concentration; Cat. No.554724). Stimulated cells were stained with PE Mouse anti-Human CD8 (Cat. No. 555367) and either PE-Cy™7 Mouse anti-Human IFN-γ (Cat. No. 557844/560741/561036, left panel) or PE-Cy™7 mouse IgG1 κ isotype control (Cat. No. 557646, right panel) by using the BD Pharmingen™ staining protocol.

Mouse Anti-human TNF, Clone MAb11, APC Flow cytometric profile of TNF expression on rhesus macaque (Macaca mulatta) peripheral blood lymphocytes. Peripheral blood lymphocytes were stimulated with PMA, then fixed, permeabilized, and washed with the BD Cytofix/Cytoperm Plus Kit (with BD GolgiStop) (Cat. No. 554715). Samples were then costained with APC Mouse Anti-Human TNF (Cat. No. 551384) and FITC Mouse Anti-Human CD3ε (Cat. No. 556611).

Anti-human CD4, clone OKT4, BV785 "FC - Quality tested, Each lot of this antibody is quality control tested by immunofluorescent staining withflow cytometric analysis. For flow cytometric staining, the suggested use of this reagent is 5 µl per million cells in 100 µl staining volume or 5 µl per 100 µl of whole blood."

Anti-human CD62L, clone DREG-56. APCcy7 "FC - Quality tested, Each lot of this antibody is quality control tested by immunofluorescent staining withflow cytometric analysis. For flow cytometric staining, the suggested use of thisreagent is 5 µl per million cells in 100 µl staining volume or 5 µl per 100 µl of wholeblood."

Anti-human PD-1, clone EH12.2H7, AF647 "FC - Quality tested, Each lot of this antibody is quality control tested by immunofluorescent staining withflow cytometric analysis. For flow cytometric staining, the suggested use of thisreagent is 5 µl per million cells in 100 µl staining volume or 5 µl per 100 µl of wholeblood."

Anti-human CD27, clone O323, BV711 FC - Quality tested Each lot of this antibody is quality control tested by immunofluorescent staining with flow cytometric analysis. For flow cytometric staining, the suggested use of this reagent is 5 µl per million cells in 100 µl staining volume or 5 µl per 100 µl of whole blood.

Anti-human CD45RA, clone HI100, BV421 FC - Quality tested, Each lot of this antibody is quality control tested by immunofluorescent staining with flow cytometric analysis. For flow cytometric staining, the suggested use of this reagent is 5 µl per million cells in 100 µl staining volume or 5 µl per 100 µl of whole blood. For immunohistochemistry, a concentration range of 5.0 - 10.0 µg/ml is suggested. It is recommended that the reagent be titrated for optimal performance for each application.

Anti-human CD45RO, clone UCHL1, AF488 FC - Quality tested, Each lot of this antibody is quality control tested by immunofluorescent staining with flow cytometric analysis. For flow cytometric staining, the suggested use of this reagent is 5 µl per million cells in 100 µl staining volume or 5 µl per 100 µl of whole blood. For immunohistochemistry, a concentration range of 5.0 - 10 µg per ml is suggested. It is recommended that the reagent be titrated for optimal performance for each application.

Anti-human CD73, Clone AD2 FC- routinely tested. This reagent has been pre-diluted for use at the recommended Volume per Test. We typically use 1 X 10e6 cells in a 100-µl experimental sample (a test).

Eukaryotic cell lines

Policy information about cell lines

Cell line source(s)	The mouse colon adenocarcinoma MC38 cell line and sarcoma 24JK cell line were kindly provided by Dr. Jeff Schlom and Dr. Patrick Hwu, respectively (National Institute of Health, Maryland, USA). The mouse breast carcinoma cell line E0771 was a gift from Prof. Robin Anderson (Olivia Newton-John Cancer Centre, Victoria, Australia). OVCAR-3, MCF7 and MDA-MB-435 were obtained from the American Type Culture Collection.
Authentication	Cell lines were not authenticated but were used within 10 passages of a master stock to ensure their accuracy
Mycoplasma contamination	All cell lines tested negative for mycoplasma contamination.
Commonly misidentified lines (See ICLAC register)	None of the cell lines used are listed on the ICLAC register.

Animals and other organisms

Policy information about studies involving animals; ARRIVE guidelines recommended for reporting animal research

Laboratory animals	C57BL/6J mice, NOD.Cg-Prkdc<scid>Il2rg<tm1Wjl> (NSG) and transgenic C57BL/6 Her2Tg mice were utilized where indicated. Studies were performed in female mice. Mice were used between 6-16 weeks of age with the exception of rechallenge experiments which necessitated the use of older mice.<br><br>Housing conditions:<br><br>Light cycle- Sunrise/sunset mode, at 6:30am they fade on for 30 mins, full lights on at 7am and fade off at 7:30pm for 30 mins, dark at 8pm. 14 hours dark. 10 hours light.<br>Temperature- 20-21C<br>Humidity- 45-60%<br>Bedding material- Crushed corncob bedding, irradiated; changed fortnightly
Wild animals	This study did not involve wild animals.
Field-collected samples	No field collected samples were used in the study
Ethics oversight	Ethics oversight was performed by the Peter MacCallum Cancer Centre Animal Experimentation Ethics Committee (AEEC).

Note that full information on the approval of the study protocol must also be provided in the manuscript.

Flow Cytometry

Plots

Confirm that:

- [x] The axis labels state the marker and fluorochrome used (e.g. CD4-FITC).
- [x] The axis scales are clearly visible. Include numbers along axes only for bottom left plot of group (a 'group' is an analysis of identical markers).
- [x] All plots are contour plots with outliers or pseudocolor plots.
- [x] A numerical value for number of cells or percentage (with statistics) is provided.

Methodology

Sample preparation	Samples from tumor-bearing mice were analyzed. Tumors were excised and digested post-mortem using a cocktail of 1mg/ml collagenase type IV (Sigma-Aldrich) and 0,02 mg/ml DNAse (Sigma-Aldrich). After digestion at 37 degrees ccelsius for 30 minutes, cells were passed through 70 micron filters twice.
Instrument	FACS data were obtained on a 5-laser FACS Symphony (Model number R660964R4002) or Fortessa (Serial number K657675J1001) (BD Pharmingen).
Software	Data were analyzed using FlowJo software (Version 10.5.0).
Cell population abundance	For experiments involving FACS sorting a purity of >90% was determined through post sort analysis.
Gating strategy	To analyze cells a preliminary FSC/SSC gate was utilized to gate on the morphology of lymphocytes. Subsequently a singles gate (FSC-A vs FSC-H) was used to exclude doublets followed by a viability gate (Fixable yellow vs CD45.2) to exclude dead cells. From this population, relevant gating strategies are described.

- [x] Tick this box to confirm that a figure exemplifying the gating strategy is provided in the Supplementary Information.
